# Regeneration of cervical reserve cell-like cells from human induced pluripotent stem cells (iPSCs): A new approach to finding targets for cervical cancer stem cell treatment

**DOI:** 10.18632/oncotarget.16783

**Published:** 2017-04-03

**Authors:** Masakazu Sato, Kei Kawana, Katsuyuki Adachi, Asaha Fujimoto, Mitsuyo Yoshida, Hiroe Nakamura, Haruka Nishida, Tomoko Inoue, Ayumi Taguchi, Juri Ogishima, Satoko Eguchi, Aki Yamashita, Kensuke Tomio, Osamu Wada-Hiraike, Katsutoshi Oda, Takeshi Nagamatsu, Yutaka Osuga, Tomoyuki Fujii

**Affiliations:** ^1^ Department of Obstetrics and Gynecology, Graduate School of Medicine, The University of Tokyo, Bunkyo-ku, Tokyo, Japan; ^2^ Department of Obstetrics and Gynecology, School of Medicine, Nihon University, Itabashi-ku, Tokyo, Japan

**Keywords:** cancer stem cell (CSC), induced pluripotent stem cell (iPSC), cervical cancer, reserve cell, human leukocyte antigen-G (HLA-G)

## Abstract

Cervical reserve cells are epithelial progenitor cells that are pathologically evident as the origin of cervical cancer. Thus, investigating the characteristics of cervical reserve cells could yield insight into the features of cervical cancer stem cells (CSCs). In this study, we established a method for the regeneration of cervical reserve cell-like properties from human induced pluripotent stem cells (iPSCs) and named these cells induced reserve cell-like cells (iRCs). Approximately 70% of iRCs were positive for the reserve cell markers p63, CK5 and CK8. iRCs also expressed the SC junction markers CK7, AGR2, CD63, MMP7 and GDA. While iRCs expressed neither ERα nor ERβ, they expressed CA125. These data indicated that iRCs possessed characteristics of cervical epithelial progenitor cells. iRCs secreted higher levels of several inflammatory cytokines such as macrophage migration inhibitory factor (MIF), soluble intercellular adhesion molecule 1 (sICAM-1) and C-X-C motif ligand 10 (CXCL-10) compared with normal cervical epithelial cells. iRCs also expressed human leukocyte antigen-G (HLA-G), which is an important cell-surface antigen for immune tolerance and carcinogenesis. Together with the fact that cervical CSCs can originate from reserve cells, our data suggested that iRCs were potent immune modulators that might favor cervical cancer cell survival. In conclusion, by generating reserve cell-like properties from iPSCs, we provide a new approach that may yield new insight into cervical cancer stem cells and help find new oncogenic targets.

## INTRODUCTION

Cancer stem cells (CSCs) are a small population of cells within tumors that possess abilities similar to normal stem cells, including the abilities to self-renew and differentiate [1–4]. This model was first documented in leukemia, and increasing evidence has suggested that this model can be applied to various types of solid tumors. Although the origin of CSCs is still controversial, it is reasonable to consider that either normal stem cells or progenitor cells that have mutated into cancer cells are the origin of CSCs [5–8].

Cervical reserve cells are generally defined as cells that are undifferentiated and act as the basal cells for columnar and squamous epithelial regeneration [9–11]. Reserve cells are located in the cervical squamocolumnar junction (SC junction). In cervical carcinogenesis, it is pathologically evident that cervical reserve cells are the origin of cervical cancer, and it is epidemiologically evident that its initiators are high-risk human papilloma viruses (HPVs) [9]. Considering these facts, investigating the characteristics of cervical reserve cells should yield valuable insight for cervical CSC research.

Female reproductive organs are derived from the Müllerian duct, which itself is derived from the intermediate mesoderm (IM) [12–15]. Indeed, previous studies that have shown regeneration of endometrial cell-like cells (i.e., the epithelial cells of the uterus) from human induced pluripotent stem cells (iPSCs) first considered inducing the IM [16]. However, to the best of our knowledge, no study has investigated either the regeneration of reserve cells from human iPSCs or the isolation and culture methods of reserve cells from primary samples.

In the present study, we present a method that we developed for the regeneration of cervical reserve cell-like properties from human iPSCs (induced reserve cell-like cells; iRCs). In addition, we suggest how these properties are potentially useful. We investigated the features of iRCs in terms of cytokine secretion patterns by using cytokine arrays. We also investigated iRC expression of human leukocyte antigen-G (HLA-G), which is involved in cervical carcinogenesis and immune tolerance but is not expressed in conventional cervical cancer cell lines at the protein level [17–19]. Here, by generating reserve cell-like properties from iPSCs, we present a new approach that may yield new insight into cervical cancer stem cell activity and function and help identify new oncogenic targets.

## RESULTS

### A small molecule-based differentiation method (the TTNPB method) produces intermediate mesoderm (IM) cells from human induced pluripotent stem cells (iPSCs)

**Figure 1:**
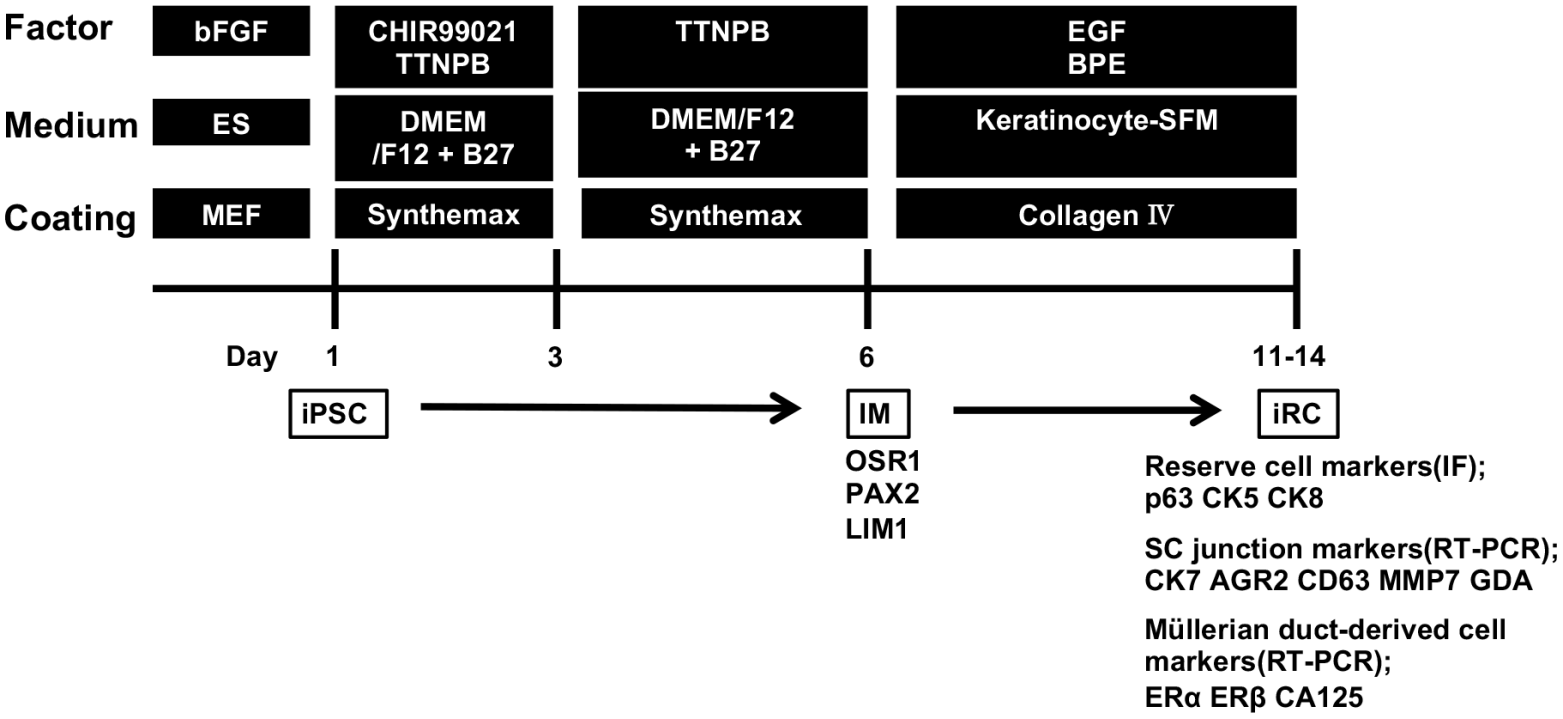
Experimental design used to induce the differentiation of human iPSCs into reserve cell-like cells (iRCs) The protocol consists primarily of two parts: the former is the induction of the IM, and the latter is the induction of epidermal cell-like cells from the IM by culturing the cells on collagen IV. bFGF, fibroblast growth factor-2; EGF, epidermal growth factor; BPE, bovine pituitary extract; MEF, mouse embryonic fibroblast; iPSC, induced pluripotent stem cell; IM, intermediate mesoderm; IF, immunofluorescence; SC junction, squamocolumnar junction.

The experimental design of this study is schematically shown in Figure 1. The TTNPB method was used for the induction of IM cells [20, 21]. In brief, human iPSCs were treated with a combination of 3 μM CHIR99021 and 1 μM TTNPB for two days followed by 1 μM TTNPB alone for an additional three days. The gene expression patterns shown in the design and the expression levels of SOX1 and SOX17 (the markers of ectoderm and endoderm, respectively) were quantified by qPCR. The expression of OSR1 was dominant (Supplementary Figure 1). Because these cells were well attached to Synthemax-coated plates, the cells were seeded on plates at a density of 1.0 to 1.5 × 10^5^ cells/cm^2^ (the literature reports slightly more cells were necessary when seeding on Matrigel). From the results above, we considered we obtained IM-like properties as described. Although it might be ideal to obtain the OSR1-GFP knock-in iPSCs and to isolate OSR1-positive cells by flow cytometry as in the literature, we proceeded to the next step.

### IM-derived cells generated with Keratinocyte-SFM on collagen IV-coated plates express reserve cell markers, squamocolumnar (SC) junction markers and the Müllerian duct-derived cell marker CA125

Although a specific marker for reserve cells has yet to be identified [22–27], we considered p63, CK5 and CK8 as reserve cell markers because they serve as markers for basal stem cells, primary squamous cells and primary glandular cells, respectively. We first investigated a method to induce populations highly expressing p63 because p63 is known to be essential for the stemness of the epithelium in the basal layers [28, 29]. In the context of inducing epithelial cells from human iPSCs, culturing cells on collagen IV-coated plates efficiently produces populations with a high percentage of p63-positive cells (80-100%) [28, 30]; therefore, we used collagen IV-coated plates. We then considered the induction medium to be used with the collagen IV-coated plates for epithelial culture and decided to use commercially available media due to their reproducibility. The investigated conditions are shown in Supplementary Figure 2. Condition (a) was described in the literature as confirming the IM cell differentiation potential used to form IM derivatives on day 14 [20, 21]. Condition (b) was used to determine the influence of the collagen IV-coating alone. Conditions (c)-(e) were candidates for further experiments to induce iRCs.

**Figure 2:**
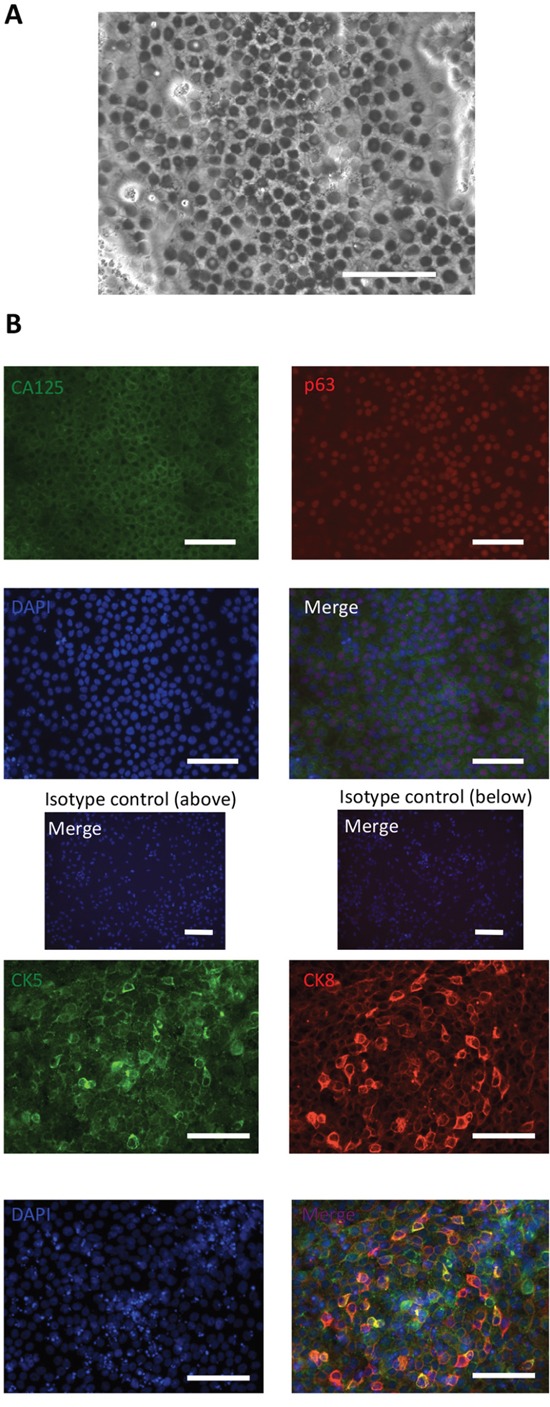
Expression of reserve cell markers of iRCs **(A)** Representative image of cells cultured in Keratinocyte-SFM medium (phase-contrast microscopy). The iRCs had round-shaped nuclei and a high nucleocytoplasmic ratio. The scale bar represents 100 μm. **(B)** Representative immunofluorescence image of iRCs on days 11-14 after differentiation. More than 70% of the iRCs were positive for the reserve cell markers. The scale bars represent 100 μm.

On day 6, IM cells were treated with 0.5 mM EDTA for 6 min and scraped off with a cell scraper. Then, these cells were centrifuged at 100 × g for 2 min and seeded onto plates under each condition at a split ratio of 1:3. The expression levels of p63 mRNA on day 14 were investigated, and condition (e) was considered to be the most efficient to induce p63 expression (Supplementary Figure 2) and was then selected for further experimental use. This result was logical because we usually use Keratinocyte-SFM medium when culturing ectocervical and endocervical cells [31]. Cells cultured under condition (e) reached almost 100% confluency near day 11 and began to float from the plate near day 14, and each marker was investigated during that period (days 11-14). Microscopic findings showed that the cells had round-shaped nuclei and a high nucleocytoplasmic ratio, which are compatible with the pathological features of reserve cells (Figure 2A). As shown in Figure 2B, these cells were confirmed to express reserve cell markers. Approximately 70% of the induced cells were positive for p63. We also investigated the expression levels of CK17 [23, 24]; the results showed that the levels were not stable and were dependent on the experimental conditions. Some of the cultures showed approximately 70% of cells expressing CK17, and others showed low expression (Supplementary Figure 3). We are unsure of what is responsible for this result; however, one reason is that identifying CK17-expressing cells during differentiation may be difficult in our selected time course.

**Figure 3:**
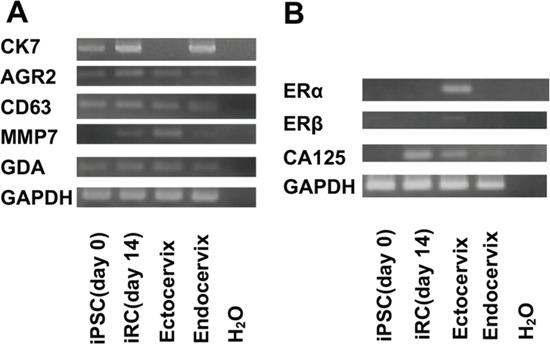
Expression of SC junction markers and Müllerian duct-derived cell markers in iRCs **(A)** Results of the RT-PCR analysis for the mRNA expression of SC junction marker genes in undifferentiated human iPSCs before treatment and in iRCs on day 14 after differentiation. The analysis showed that the iRCs and endocervical cells expressed each marker. **(B)** Results of the RT-PCR analysis for the mRNA expression of Müllerian duct-derived cell marker genes in undifferentiated human iPSCs before treatment and in iRCs on day 14 after differentiation. The analysis showed that iRCs and endocervical cells did not express either ERα or ERβ but expressed CA125. SC junction; squamocolumnar cell junction.

These cells also expressed SC junction markers (Figure 3). While these cells expressed neither ERα nor ERβ, they expressed CA125, which is discussed later in this article (Figure 3).

### IRCs form glandular epithelial-like cells and stratified squamous epithelial-like cells in three-dimensional cultures

**Figure 4:**
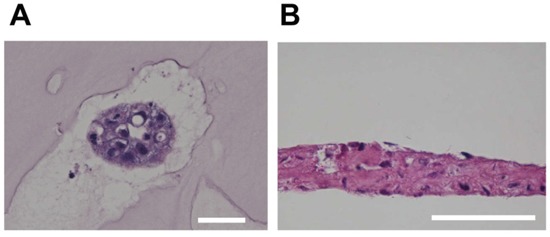
H&E staining of iRCs in three-dimensional cultures **(A)** Representative image of iRCs in the 3D-embedded culture. The iRCs formed glandular epithelial cell-like cells within two to three days. Note that they formed an inner cavity, unlike the spheroids from cancer cells. The scale bars represent 50 μm. **(B)** Representative image of iRCs in an air-liquid interface culture after one to three weeks. The iRCs were pseudostratified rather than properly stratified. The scale bars represent 50 μm.

A specific feature of reserve cells is bi-potency, or the ability to differentiate into either glandular epithelium or stratified squamous epithelium [2, 23]. To confirm the potency of iRCs, we applied three-dimensional culture methods as described in the Materials and Methods. 3D-Embedded cultures are usually used for cultures of mammary, prostate and salivary glandular epithelial cells [32–35]. A cavity was found inside the 3D-embedded cultures unlike the spheroids that are generally formed from cancer cells. Air-liquid interface cultures were used for culturing skin and corneal epithelial cells [28, 36]. As shown in Figure 4, iRCs formed glandular epithelial-like cells in the 3D-embedded culture. For the air-liquid interface culture, the iRCs were pseudostratified rather than properly stratified (Figure 4), which was also observed in the respiratory epithelial cell culture [37].

### IRCs are involved in inflammation by secreting inflammatory cytokines

**Figure 5:**
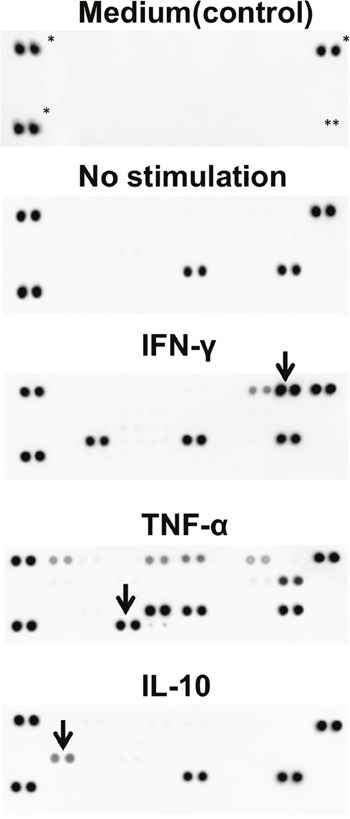
Cytokine secretion patterns of iRCs The detected cytokines under each condition were as follows: no stimulation, MIF and PAI1; IFN-γ, MIF, PAI1, sICAM1 and CXCL10; TNF-α, MIF, PAI1, C5a, GM-CSF, CXCL1, IFN-γ, IL-8 and CCL2; IL-10, MIF and PAI1 (i.e., no response). *: positive control, **: negative control, arrows: recombinant cytokines used for stimulation. MIF; macrophage migration inhibitory factor, PAI1; plasminogen activator inhibitor-1, sICAM; soluble intercellular adhesion molecule, CXCL; C-X-C motif ligand, IFN; interferon, TNF; tumor necrosis factor, GM-CSF; granulocyte macrophage colony-stimulating factor, CCL; chemokine ligand.

Various types of infection occur in the uterine cervix [38, 39]. In fact, ectocervical and endocervical epithelial cells are known to be involved in the inflammation of the cervix [40]. We speculated whether cervical reserve cells are also involved in inflammation. Upon performing a cytokine array analysis, we detected cytokines in the iRC supernatants with or without stimulation of interferon (IFN)-γ, tumor necrosis factor (TNF)-α and IL-10. As shown in Figure 5, iRCs normally secrete plasminogen activator inhibitor-1 (PAI-1) and macrophage migration inhibitory factor (MIF). After stimulation with IFN-γ and TNF-α, the iRCs secreted various inflammatory cytokines such as soluble intercellular adhesion molecule (sICAM)-1 and C-X-C motif ligand (CXCL)-10 (Figure 5). These patterns were not identical to the cytokine secretion from conventional ectocervical and endocervical cancer cell lines [40].

### IRCs express human leukocyte antigen-G (HLA-G) highly

**Figure 6:**
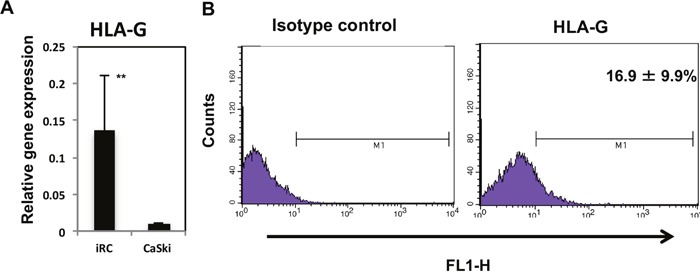
HLA-G expression in iRCs **(A)** The expression level of HLA-G mRNA in iRCs and CaSki cells (cervical cancer cells). The iRCs expressed significantly higher levels of HLA-G mRNA than the CaSki cells. **(B)** Results of the flow cytometric analysis for HLA-G-positive cells in iRCs on day 14 after differentiation. The data are presented as the means ± S.Ds. of three independent experiments. **, p < 0.01. HLA-G, human leukocyte antigen-G.

HLA-G is related to cervical cancer carcinogenesis [19]. Most cervical cancer cell lines, however, barely express HLA-G at the mRNA level and minimally express HLA-G on the cell surface, which makes studying HLA-G more complicated [41]. We investigated the HLA-G expression levels in iRCs and found that iRCs expressed significantly higher levels of HLA-G mRNA than the cervical cancer cell line CaSki (p = 0.0032). We also confirmed that iRCs expressed HLA-G on the cell surface by using flow cytometry (Figure 6).

## DISCUSSION

Here, we showed the regeneration of cervical reserve cell-like properties from human iPS cells (iRCs) and the potential utility of this process for cervical CSC research.

CSCs are a small population of cells within tumors that possess abilities similar to normal stem cells. Although the origin of CSCs is still an ongoing research topic, it has been accepted that CSCs can originate from either normal stem cells or progenitor cells [3–5]. Cervical cancer is quite distinctive from other types of solid tumors in that its origin and initiators are obvious—it is pathologically evident that cervical reserve cells, which are located in the cervical SC junction and which possess bi-potency to become squamous and columnar epithelial cells, are the origin of cervical cancer, and it is epidemiologically evident that their initiators are high-risk HPVs [9–11].

First, we demonstrated the regeneration of induced reserve cell-like cells (iRCs). Approximately 70% of iRCs are positive for the reserve cell markers p63, CK5 and CK8. IRCs also express the SC junction markers, CK7, AGR2, CD63, MMP7 and GDA. Female reproductive organs are derived from the Müllerian duct, and Müllerian duct-derived cells in adult tissues are thought to express ERα, ERβ and CA125. While the iRCs expressed neither ERα nor ERβ, they expressed CA125. This finding was compatible because ERα is not needed during uterus development, and the neonatal cervix does not express ERα [42, 43]. Indeed, the endometrium regenerated from iPSCs did not express ERα but did express CA125 [16]. Considering that the iRCs were positive for p63, we may conclude that the properties we induced resembled cells residing in the uterine cervix. Accordingly, the gene expression patterns of the iRCs were similar to those of endocervical cells (Figure 3), which was acceptable because reserve cells exist beneath endocervical cells and are thought to have similar features [22]. We speculated the properties that did not express the reserve cell marker p63. Some were cells which did not express p63 but expressed cytokeratin 5 or 8, which we thought were relatively differentiated cells. In addition, we found that some were shaped like fibroblasts and others looked relatively undifferentiated. In other words, the populations existed which had differentiated toward unwanted directions, as is often the case with iPSCs differentiation. And our protocol itself needs refinement in terms of efficiency.

Second, we investigated the features of iRCs in terms of tumor immunity. The iRCs secreted inflammatory cytokines when exposed to IFN-γ and TNF-α. This result was contrary to our first expectation because stem cells are thought to have some immune tolerance, and we sought factors related to immune escape. Then, we found that iRCs highly expressed HLA-G at both the mRNA and protein levels. HLA-G is a member of the human leukocyte antigen (HLA) family and is involved in inhibiting the immune response [44, 45]. These observed data led us to speculate that reserve cells tactically maintain the balance between anti- and pro-inflammatory functions.

One limitation of our study was that we did not have any positive controls for the reserve cells. Because there is no definition for reserve cells, for example, in terms of gene expression patterns or gene expression levels, we could not demonstrate that the properties we induced were really reserve cells [10, 22–24, 27]. However, we showed a new approach for cervical CSC research, and in that sense, we can conclude that iRCs are potentially useful. Indeed, we focused on the relationship between cervical CSCs and PAI-1 from the cytokine analysis data (Figure 5), and, using the conventional cervical cancer cell lines CaSki and SiHa, we have already shown that PAI-1 plays a role in the maintenance of the extracellular matrix surrounding cervical CSCs [46]. In addition, we found the discrepancies between extracellular HLA-G expression and intracellular HLA-G expression. For instance, percentages of extracellular HLA-positive cells were about 10% on day 6 (Supplementary Figure 4A), while percentages of intracellular HLA-positive cells were about 76% (Supplementary Figure 4B). These results were compatible with previous reports [47, 48]. One group demonstrated that most of the Nanog-positive human embryonic stem cells were also positive for intracellular HLA-G [47]. Other group investigated the expression of HLA-G in human bone marrow-derived mesenchymal stem cells, and reported that percentages were 22% for extracellular HLA-G and 61% for intracellular HLA-G [48]. We assumed that these findings are attributable to the expression levels of transporter associated with antigen processing 1(TAP 1 or ABCB2) [49]. Indeed, the expression of TAP1 preceded that of HLA-G during the course of inducing iRCs (Supplementary Figure 4C). And we further speculated that the high expression of TAP1 could be one of cancer stem cell-like cells features. To test this, we compared the expression levels of TAP1 between cancer stem cell-like properties (e.g., spheroids) and adherent cells, using cervical cancer cell lines, HeLa, CaSki and SiHa. And we found that the expression levels of TAP1 of spheroids were higher than those of adherent cells (Supplementary Figure 4D). These results are preliminary, and the translational importance is still limiting. However, we might say that using iRCs or iPSCs could be a good model and method to investigate the characteristics of HLA-G, and to obtain insight into cervical cancer stem cells.

The advantage of inducing cervical reserve cells from human iPSCs is that this process genuinely reflects development and carcinogenesis unlike other models such as culturing iPSCs with cancer cells and introducing Yamanaka factors to the cancer cells [50–52]. Indeed, we are now studying how to immortalize iRCs with HPV E6 and E7 infections, and we are characterizing the cells via cell expansion and single-cell separation.

In conclusion, we propose a new approach for cervical CSC research involving the generation of cervical reserve cell-like cells from iPSCs. Although our protocol itself needs refinement in terms of efficiency, this study introduces an approach based on iPSCs that can be used in studies of cancer stem cells in multiple fields.

## MATERIALS AND METHODS

### Cell culture

The human iPS cell line 201B7 was provided by the RIKEN BRC through the National Bio-Resource Project of the MEXT (Japan) and cultured as described in the literature [53]. In brief, iPS cells were cultured on feeder layers of mitomycin C-treated mouse embryonic fibroblasts (MEFs) in primate ES medium supplemented with 5 ng/ml recombinant basic fibroblastic growth factor (bFGF) and sub-cultured using a dissociation solution at a split ratio of 1:4 every 4 days. The medium was changed daily. MEFs were seeded onto plates coated with ReproCoat and cultured in Dulbecco's modified Eagle medium (DMEM; Wako, Japan) supplemented with 10% fetal bovine serum (FBS; Invitrogen, USA). All reagents mentioned above were purchased from ReproCELL (Japan) unless otherwise stated. All cells were cultured in a humidified atmosphere at 37 °C and 5% CO_2_.

The induction of IM cells from iPS cells was performed as described [20, 21]. In short, after removing the MEFs with the ReproCoat-coated plates, the iPS cells were seeded into Synthemax (Synthemax II-SX Substrate, Corning, USA)-coated 6-well plates in DMEM/F12 + Glutamax supplemented with 1× B27 supplement (Invitrogen, USA) and 500 U/ml penicillin/streptomycin (Wako). On day 1, 10 μM Y27632 (Wako, Japan) was added. The cells were treated with 3 μM CHIR99021 and 1 μM TTNPB (Wako) for two days, and the culture medium was replaced with medium containing 1 μM TTNPB and maintained for an additional three days.

For induction of iRCs, the following commercially available media were obtained and compared: CnT basal medium, CnT-Prime medium, and CnT PD medium were purchased from CELLnTEC (Switzerland), and Keratinocyte-SFM was purchased from Invitrogen. CnT basal medium was supplemented with 20 ng/ml epidermal growth factor (EGF; Wako). Collagen IV-coated 6-well plates (Corning) were used except in instances that required inserts (pore size 0.4 μm, Corning), which were coated with human placental collagen IV (10 μg/cm^2^, Sigma-Aldrich, USA).

### Three-dimensional culture

3D cultures were performed using two methods. One included an air-liquid interface, for which induced intermediate mesoderm cells were seeded into 35-mm inserts (coated with collagen IV) in one well of a 6-well plate [54]. After reaching confluence, the medium (Keratinocyte-SFM) inside the inserts was removed, and the cells were exposed to the air for one to three weeks.

The other method was a 3D-embedded culture in which cells were dissociated on day 10 and seeded into ultra-low attachment 24-well plates (Corning) using CnT-Prime 3D Barrier medium (CELLnTEC) with 10 μM ROCK inhibitor for one day; this procedure allowed cells to form spheroids. All of the spheroids were then embedded into Matrigel (Corning)-coated plates prepared as follows. One well of the 6-well plates was coated with 250 μl of Matrigel and incubated at 37 °C for 2 h before use (which induced the Matrigel to form a semisolid). The medium used was a mixture of 250 μl of Matrigel (liquid) and 250 μl of CnT-Prime 3D Barrier medium. Within 2 to 3 days, the cells inside the spheroids were differentiated, and apoptosis was induced resulting in the formation of glandular cell-like cells.

### RT-PCR and real-time quantitative RT-PCR (qPCR)

Total RNA was extracted using a Tissue Total RNA Kit (Favorgen Biotech Corp., Taiwan) according to manufacturer's recommendations. First-strand cDNA was synthesized from 500 ng of total RNA using ReverTra Ace (Toyobo, Japan).

qPCR was performed with SYBR Green PCR master mix (Roche) according to the manufacturer's instructions. Denaturation was performed at 95 °C for 2 min followed by 35 cycles at 98 °C for 10 s, 65 °C for 10 s and 68 °C for 8 s. β-actin was used as an internal control, and the results are presented as the fold-change relative to β-actin expression (2^-ΔΔCt^). Each experiment was performed in triplicate.

See Supplementary Table 1 for the primer list.

### Immunofluorescence and immunohistochemistry

Immunofluorescence was performed as previously described [28]. iRCs on days 11 to 14 were fixed with 4% paraformaldehyde for 20 min and washed three times with PBS. Cell membranes were then permeabilized for 10 min with 0.1% Triton X-100. Nonspecific binding sites were blocked with 3% BSA for 1 h. Primary antibodies were diluted in 1% BSA and 0.3% Triton X-100 and incubated overnight at 4 °C. Primary antibody detection was performed using the appropriate Alexa Fluor 488- or 594-conjugated secondary antibodies diluted in 1% BSA and 0.3% Triton X-100 for 1 h at room temperature (See Supplementary Table 2 for a list of the primary and secondary antibodies). Finally, the cells were stained with 4',6-diamidino-2-phenylindole (DAPI) for visualization of the nuclei. Images of the stained cells were captured using a BZ-X700 fluorescence microscope (Keyence, Japan). The total cell number was determined by counting the DAPI-stained nuclei using hybrid cell counting BZ-H3C software (Keyence), and the percentage of positive cells for each reserve cell marker was quantified. Areas with adherent cells were randomly selected, and the cells were counted. This analysis was repeated at least three times. Each area contained an average of 302 cells. At least three independent experiments were performed on different days.

To make a paraffin block of the cell suspension (3D-embedded culture), iPGell (Genostaff, Japan) was used. To make the cell blocks, prepared cell suspensions were solidified with iPGell according to the manufacturer's instructions. The cell blocks were fixed with G-Fix (Genostaff), embedded in paraffin on a CT-Pro20 (Genostaff) using G-Nox (Genostaff) as a less toxic organic solvent in place of xylene, and sectioned at a thickness of 5-6 μm.

For H&E staining, tissue sections were deparaffinized with xylene, rehydrated using a series of ethanol concentrations, and rinsed in running tap water for 5 min. The sections were treated with hematoxylin (Sigma) for 10 min, rinsed in running tap water for 20 min, and finally treated with eosin alcohol (Sigma) for 5 min. The sections were dehydrated using a series of ethanol solutions and xylene and then mounted with Malinol (Muto, Japan).

### Cytokine array analysis

A cytokine array analysis (R&D system, Cat# ARY005) was performed according to the manufacturer's instructions. The concentration of each cytokine for stimulation was as follows: IFN-γ (Wako), 20 ng/ml; TNF-α (Wako), 20 ng/ml and IL-10 (Wako), 50 ng/ml [40]. The experiments for each stimulation were repeated at least twice, and similar results were obtained.

### Flow cytometry

Flow cytometric analysis was performed by staining 1-2 × 10^7^ iRCs using an FACSCalibur flow cytometer (BD Biosciences, Japan) [55]. In brief, cells were stained with the mouse anti HLA-G FITC antibody for 30 minutes at 4 °C. Control aliquots were stained with an isotype-matched antibody (Biolegend, Cat#400110) to evaluate nonspecific binding to target cells. Intracellular staining was performed using the Cytofix/Cytoperm (BD Biosciences) as previously described [47]. The experiment was repeated three times. See Supplementary Table 2 for the antibodies used.

### Statistical analysis

Student's t-tests (two-tailed) were used to compare the means. P values less than 0.05 were considered statistically significant. The JMP^®^/SAS Institute was used for the statistical analysis.

## SUPPLEMENTARY MATERIALS FIGURES AND TABLES


