# Supplementary material for: Regeneration of cervical reserve cell-like cells from human induced pluripotent stem cells (iPSCs): A new approach to finding targets for cervical cancer stem cell treatment
Source: Oncotarget. 2017 Apr 3;8(25):40935–45. doi: 10.18632/oncotarget.16783 (PMC5522215; doi:10.18632/oncotarget.16783)
Supplement: Supplementary file 1 [file oncotarget-08-40935-s001.pdf]

Regeneration of cervical reserve cell-like cells from human induced pluripotent stem cells (iPSCs): A new approach to finding targets for cervical cancer stem cell treatment

SUPPLEMENTARY FIGURES AND TABLES

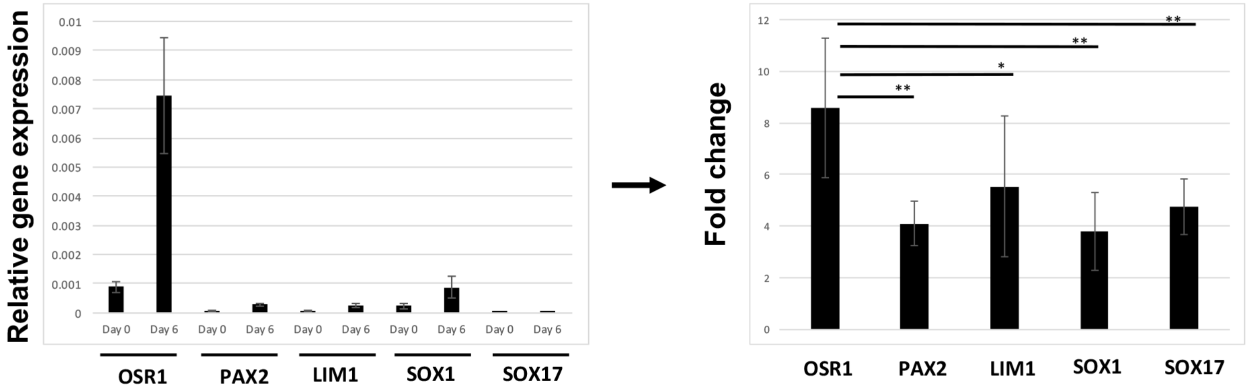

Supplementary Figure 1: Expression of each marker of cells on day 0 (iPSCs) and on day 6 after induction by qPCR. The gene expression levels of OSR1, PAX2, LIM1 (i.e., markers of IM), SOX1 (a marker of ectoderm), and SOX17 (a marker of endoderm) were quantified by qPCR (Left). The expression levels were normalized to that of the housekeeping gene β-actin. Fold changes from day 0 to day 6 for each gene expression were investigated, and the expression of OSR1 was dominant (paired t-test, Right). The data are presented as the means ± S.D. of three independent experiments. *, $p < 0.05$; **, $p < 0.01$. IM, intermediate mesoderm.

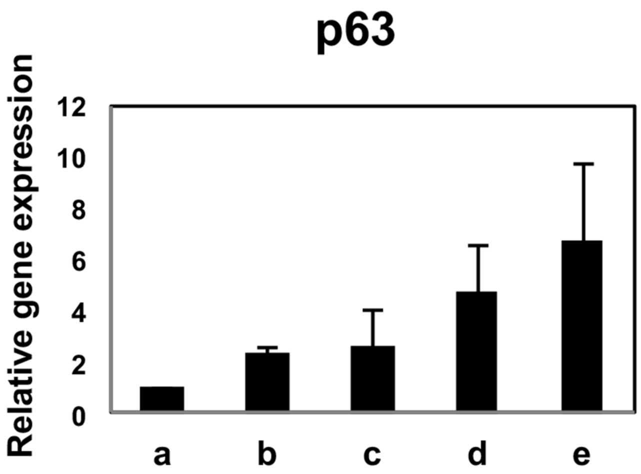

Supplementary Figure 2: qPCR screening strategy of determining an efficient protocol to induce cells that highly express p63. The investigated (i) medium, (ii) growth factors and (iii) types of coatings were as follows. (a) (i) DMEM/F12+Glutamax supplemented with 1 × B27, (ii) 100 ng/ml BMP7 and 100 ng/ml Wnt3a, and (iii) Synthemax; (b) (i) DMEM/F12+Glutamax supplemented with 1 × B27, (ii) none, and (iii) collagen IV; (c) (i) CnT-Prime Basal medium, (ii) 20 ng/ml EGF, and (iii) collagen IV; (d) (i) CnT-Prime Differentiation medium, (ii) none, and (iii) collagen IV; and (e) (i) Keratinocyte-SFM, (ii) 20 ng/ml EGF and BPE (provided with the medium), and (iii) collagen IV. Condition (e) was considered to be the most efficient to induce p63 expression. The data are presented as the means ± S.D. of three independent experiments.

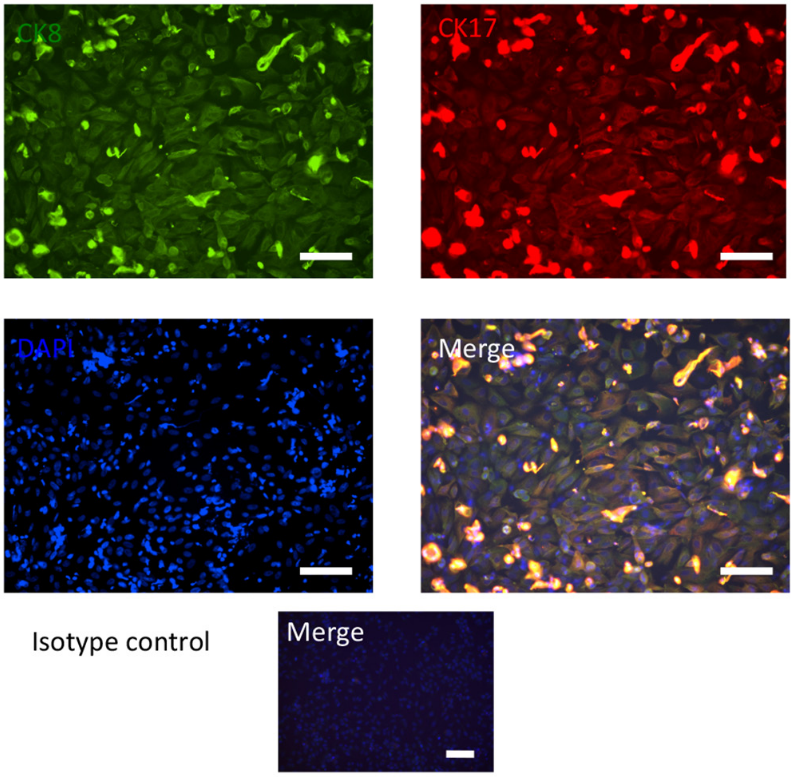

Supplementary Figure 3: Expression of CK17 of iRCs. Immunofluorescence image of iRCs on day 14 after differentiation. Approximately 70% of the iRCs were positive for CK17 in this experiment. However, we found that this expression was not stable and was dependent on the individual experiments. Some of the cultures reached approximately 70% expression, but others showed low expression. The scale bars represent 100 µm.

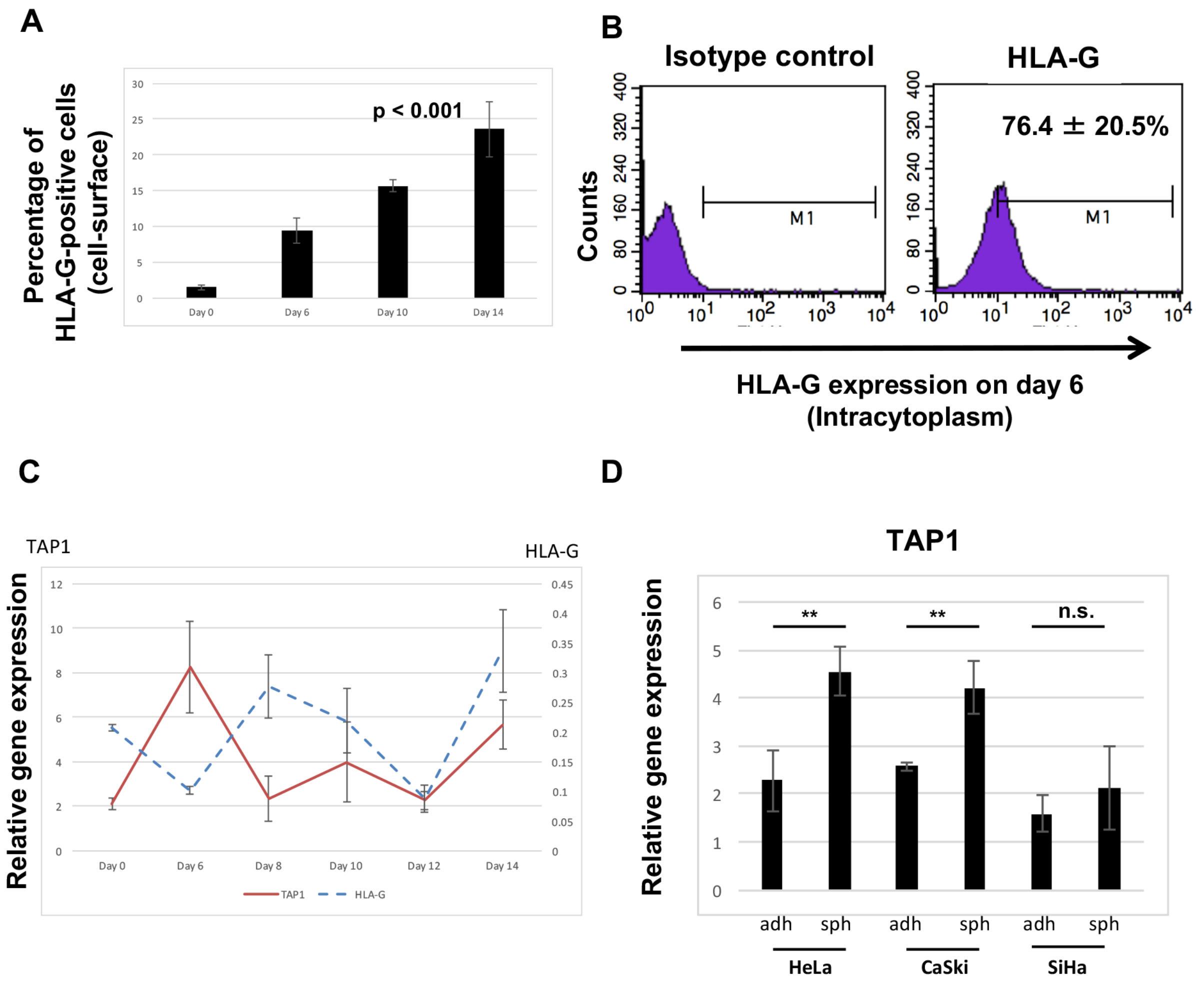

Supplementary Figure 4: Investigation of HLA-G and TAP1. (A) Percentage of extracellular HLA-positive cells during differentiation. Percentages of extracellular HLA-positive cells gradually but significantly increased during differentiation (ANOVA). The data are presented as the means ± S.D. of three independent experiments. **(B)** Representative result of the flow cytometric analysis for intracellular HLA-G-positive cells on day 6 after differentiation. Intracellular HLA-G antigens were detected after cell permeabilization. The data are presented as the means ± S.D. of three independent experiments. **(C)** Expression of TAP1 and HLA-G after induction by qPCR. The expression of TAP1 peaked on day 6, while the expression of HLA-G got up and down. The data are presented as the means ± S.D. of three independent experiments. Y-axis were relative gene expression normalized to the expression of β-actin, which was multiplied by 1000 for improving visualization. **(D)** Expression of TAP1 by qPCR in cervical cancer cell lines. The expression levels of TAP1 of spheroids were higher than those of adherent cells. Y-axis were relative gene expression normalized to the expression of β-actin, which was multiplied by 1000 for improving visualization. **, $p < 0.01$; n.s., not significant. HLA-G, human leukocyte antigen-G; TAP1, transporter associated with antigen processing 1; adh, adherent cells; sph, spheroids.

Supplementary Table 1: Primer sequences used in this study

Gene name	Primer sequence	Size (bp)
Intermediate mesoderm		
hOSR1	GCTGTCCACAAGACGCTACA<br>CCAGAGTCAGGCTTCTGGTC	137
hPAX2	GCTTTGGATCGGGTCTTTGA<br>CTCGTTCCCCTGTTCTGATTTG	84
hLIM1	TCATGCAGGTGAAGCAGTTC<br>TCCAGGGAAGGCAAACTCTA	148
Ectoderm		
hSOX1	CACAACTCGGAGATCAGCAA<br>GGTACTTGTAATCCGGGTGC	133
Endoderm		
hSOX17	GGCGCAGCAGAATCCAGA<br>CCACGACTTGCCCAGCAT	61
Reserve cell		
p63	TCCTCAGGGAGCTGTTATCC<br>ATTCACGGCTCAGCTCATGG	101
SC junction		
CK7	TCCGCGAGGTCACCATTAAC<br>GCTCTGTCAACTCCGTCTCAT	519
AGR2	GTCAGCATTCTTGCTCCTTGT<br>GGGTCGAGAGTCCTTTGTGTC	97
CD63	ATGCAGGCAGATTTTAAGTGCT<br>GTTCTTCGACATGGAAGGGATTT	75
MMP7	GAGTGAGCTACAGTGGGAACA<br>CTATGACGCGGGAGTTTAACAT	158
GDA	GCTGGAAGTAGCATAGACCTGC<br>TCTTCTGCAAAGTCGATGTTCTG	95
Müllerian duct-derived cell		
ERα	CCCACTCAACAGCGTGTCTC<br>CGTCGATTATCTGAATTTGGCCT	180
ERβ	CCGACAAGGAGTTGGTACA<br>CAGGAGCATCAGGAGGTTA	482
CA125	GCCTCTACCTTAACGGTTACAATGAA<br>GGTACCCCATGGCTGTTGTG	114
Housekeeping genes and HLA-G		
β-actin	CATGTACGTTGCTATCCAGGC<br>CTCCTTAATGTCACGCACGAT	250
GAPDH	GAAAGGTGAAGGTCGGAGTC<br>GAAGATGGTGATGGGATTTC	227
HLA-G	CACGCACAGACTGACAGAATG<br>GCCATCGTAGGCATACTGTTCA	153

Gene names and primer sequences(5’-3’) for RT-PCR and qPCR are shown.

Supplementary Table 2: Antibodies used in this study

Antibody	Host	Dilution	Manufacturer and cat#
p63	Mouse	1:100	abcam, ab735
CK8	Mouse	1:100	Santa Cruz Biotechnology, sc-8020
CK5	Rabbit	1:500	abcam, ab24647
CK17	Rabbit	1:100	abcam, ab109725
CA125(MUC16)	Rabbit	1:100	abcam, ab134093
Alexa Fluor 488 anti-mouse	Rabbit	1:800	Molecular Probes, A-11059
Alexa Fluor 488 anti-rabbit	Goat	1:800	abcam, ab150077
Alexa Fluor 594 anti-mouse	Goat	1:800	Molecular Probes, A-11005
Alexa Fluor 594 anti-rabbit	Goat	1:800	Molecular Probes, A-11012
HLA-G (FITC)	Mouse	1:500	abcam, ab7904

Primary and secondary antibodies used for characterization of iRCs.
